# A Cell Electrofusion Chip for Somatic Cells Reprogramming

**DOI:** 10.1371/journal.pone.0131966

**Published:** 2015-07-15

**Authors:** Wei Wu, Ya Qu, Ning Hu, Yuxiao Zeng, Jun Yang, Haiwei Xu, Zheng Qin Yin

**Affiliations:** 1 Southwest Eye Hospital, Southwest Hospital, Third Military Medical University, Chongqing, P.R. China; 2 Key Lab of Visual Damage and Regeneration & Restoration of Chongqing, Chongqing, P.R. China; 3 Bioengineering College of Chongqing University, Chongqing, P.R. China; Friedrich-Loeffler-Institute, GERMANY

## Abstract

Cell fusion is a potent approach to explore the mechanisms of somatic cells reprogramming. However, previous fusion methods, such as polyethylene glycol (PEG) mediated cell fusion, are often limited by poor fusion yields. In this study, we developed a simplified cell electrofusion chip, which was based on a micro-cavity/ discrete microelectrode structure to improve the fusion efficiency and to reduce multi-cell electrofusion. Using this chip, we could efficiently fuse NIH3T3 cells and mouse embryonic stem cells (mESCs) to induce somatic cells reprogramming. We also found that fused cells demethylated gradually and 5-hydroxymethylcytosine (5hmC) was involved in the demethylation during the reprogramming. Thus, the cell electrofusion chip would facilitate reprogramming mechanisms research by improving efficiency of cell fusion and reducing workloads.

## Introduction

Differentiated somatic cells can be reprogrammed into pluripotent stem cells by nuclear transfer into enucleated oocytes [1], co-culture with stem cell extract [2], transcription factor transduction [3] or by cell fusion [4], which has a great prospect in regenerative medicine. Through years of researches, it is shown that reprogramming is influenced by the DNA methylation status [4–6]. Ten-Eleven Translocation (TET) enzymes can convert 5-methylcytosine (5mC) to 5hmC or further oxidize 5hmC to 5-formylcytosine (5fC) and 5-carboxylcytosine (5caC) [7–9]. 5hmC, which is a new epigenetic marker, plays a crucial role in DNA demethylation [10, 11]. To advance the clinical application of induced pluripotent stem cells (iPSCs) and further elucidate reprogramming mechanisms, a multitude of studies focus on enhancing reprogramming efficiency and speed. Cell fusion has been demonstrated to be a potent way of illuminating the mechanisms of somatic cells reprogramming due to its high efficiency and celerity [12]. Although PEG is notoriously inefficient and toxic [13], it is still the most commonly utilized cell fusion reagent to study reprogramming mechanisms because PEG is easy-to-get. Besides, traditional electrofusion method is also applied in reprogramming research occasionally [14, 15]. However, the traditional electrofusion method is also inefficient and the resultant high Joule heating will impair fused cells [16].

Recently, microfluidic chip-based cell electrofusion has shown great potential [13, 17], due to its high fusion efficiency and precise manipulation ability. In addition, this system also reduces the working voltage and the negative effect of Joule heating. Electrofusion is accomplished after two processes, cell pairing and cell electrofusion. Since dielectrophoretic (DEP) force is safe and easy to operate, DEP force-based cell pairing is extensively considered [18–21]. To improve the heterogeneous cell pairing, microstructures for cell capture and cell pairing are integrated on microfluidic devices. Cells can be captured and paired in the microstructures, with hydrodynamic [22, 23], DEP [24, 25], or chemical interactions control [13]. To further improve electrofusion efficiency, microelectrodes geometry modification and electric field constriction are used to optimize electric field [13, 17–19, 23–25]. More recently, nanopulses-based electroporation attracts great attention, due to the high electroporation efficiency and robust cell survival [26]. It shows great application potential in cell electrofusion.

Previously, we have developed a microfluidic chip for high throughput cell electrofusion, which has a dense microelectrode array for the simultaneous pairing and electrofusion of thousands of cells by manipulating the DEP force and electroporation [27]. Here, we designed and fabricated a new microfluidic device based on thousands of micro-cavity/ discrete microelectrode structures to improve cell pairing/ electrofusion efficiency and to reduce multi-cell electrofusion. Compared with the previous chip, the space area between two adjacent microelectrodes was filled by insulated floating silicon to avoid cells pairing in this area where electric field was not enough to induce cell electrofusion. In addition, this design could concentrate electric field to induce reversible electroporation. Using this microfluidic chip, we could efficiently electrofuse NIH3T3 cells and mESCs to induce NIH3T3 cells reprogramming. The pluripotency of these electrofused cells and the mechanisms of reprogramming mediated by electrofusion were explored.

## Materials and Methods

### Ethics Statement

The nude mice used in this research were obtained from the Third Military Medical University and were maintained at pathogen-free conditions. All procedures were done according to protocols approved by the Institutional Review Board of the Southwest Hospital, Third Military Medical University and conformed to the NIH guidelines on the ethical use of animals.

### Design and fabrication of cell electrofusion chip

**Fig 1:**
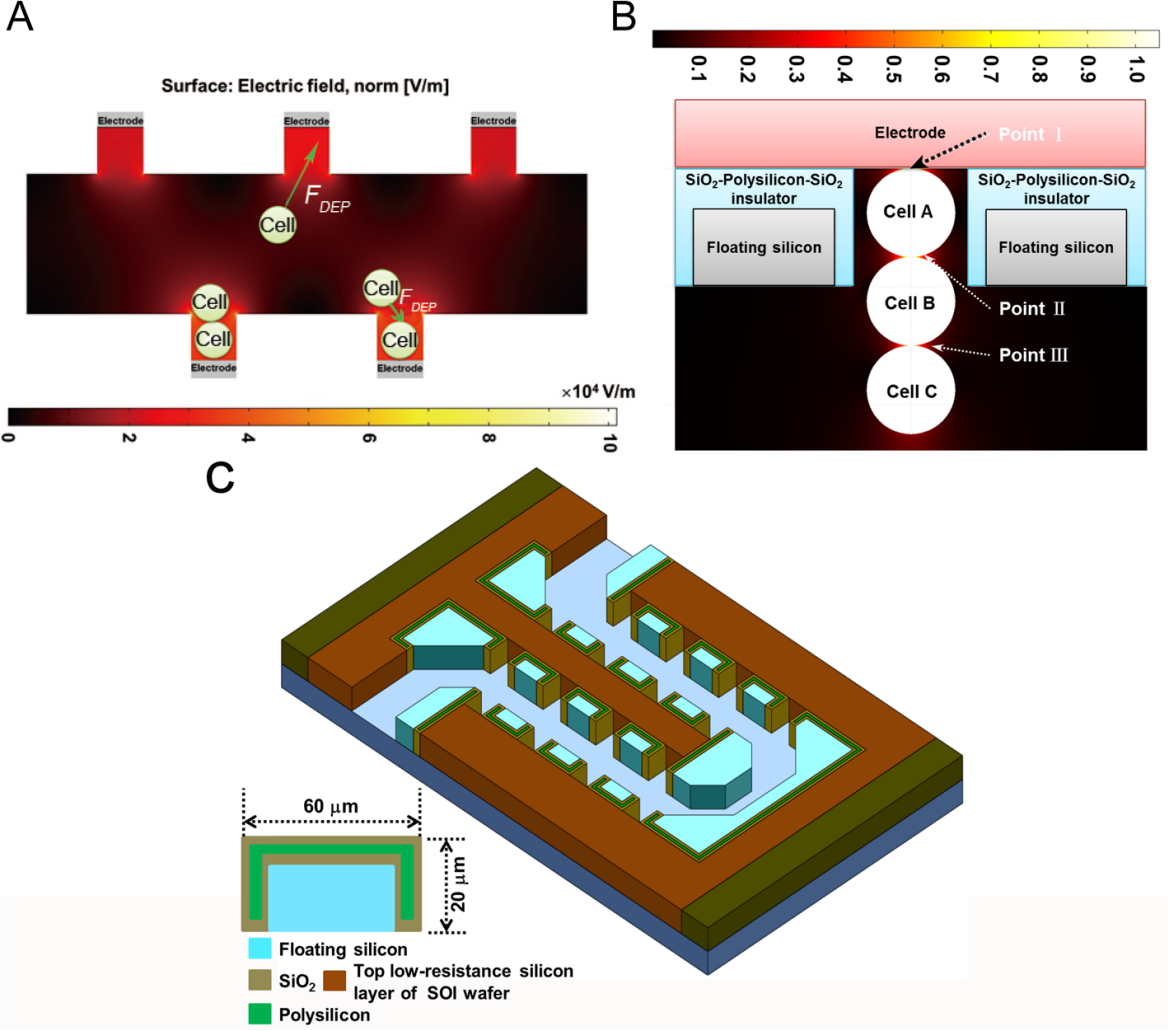
The structure and electric field distribution of cell electrofusion chip. (A) Cell trapping and pairing under positive-DEP force. (B) The electric field distribution in the cell electrofusion chip. (C) A 3D schematic of the cell electrofusion chip based on micro-cavity/ discrete microelectrode structure.

As shown in Fig 1C, this microfluidic chip consisted of two chiasm-shaped microelectrode arrays, which was fabricated on a SOI wafer. To provide good mechanical support for this microfluidic chip, we chose a SOI wafer with 430 μm thickness base silicon layer. And the buried SiO_2_ layer ensured desired electrical insulation. The two chiasm microelectrode arrays and serpentine-shaped microfluidic channel were fabricated by etching 35 μm thick top low-resistance silicon layers. On each microelectrode arrays, approximate 1.9×10^4^ micro-cavity/ discrete microelectrode structures were integrated. In each micro-cavity structure, the exposure low-resistance silicon sidewall that was parallel to the microchannel served as a microelectrode, whereas the other two sidewalls, which were perpendicular to the microchannel, were fabricated by a SiO_2_ insulator. Since the separation distance between two adjacent micro-insulators was 20 μm, the width of microelectrode (exposure silicon between two adjacent micro-insulators) was also 20 μm. Each SiO_2_-Polysilicon-SiO_2_ micro-insulator was 60 μm in length and 20 μm in width. And it was composed of 600 nm thick SiO_2_ insulator wall, a 1.8 μm thick enclosing ploysilicon wall, which provided mechanical support [24]. In addition, the floating silicon, which was enclosed by the SiO_2_-Polysilicon-SiO_2_ micro-insulator, provided the sidewall of serpentine-shaped microfluidic channel.

After fabrication process, this microfluidic chip was bonded with a PDMS cover. According to the inlet and outlet fluid reservoirs in the microfluidic chip, the PDMS cover contained inlet and outlet holes (diameter: 2mm). In addition, it also required the electrical connection with power generator. By bonding technology, the connection between microfluidic chip and printed circuit board (PCB) was created with gold silk (diameter: 75 μm). The dimensions of the entire chip were 3 cm (length) ×2 cm (width) ×470 μm (thickness).

### Cell culture

NIH3T3 cells originated from American Type Cell Culture and were transfected with lentiviral vector pZLENU-tagRFP. As previously described, GFP^+^ mESCs originated from a mouse embryonic stem cell, MESPU35, which stably expressed GFP [28]. Red fluorescent protein (RFP) positve NIH3T3 cells were cultured in High glucose Dulbecco’s modified Eagle’s medium (H-DMEM; HyClone) containing 4500 mg/L glucose supplemented with 10% fetal bovine serum (FBS; HyClone). GFP^+^ mESCs were cultured with irradiated embryonic fibroblasts (Cyagen Biosciences) as the feeder. Culture media for mESCs (Cyagen Biosciences) contained H-DMEM, which was supplemented with 10% FBS, 2 mM L-glutamine, 100 μg/ml penicillin–streptomycin, 1000 IU/ml leukemia inhibitory factor, 0.1 mmol/l β-mercaptoethanol, and 0.1 mmol/l nonessential amino acids. Fused cells were cultured in the identical culture media as GFP^+^ mESCs without feeder.

### Cell electrofusion and isolation by flow cytometry

The cell electrofusion was performed as described previously [27]. In brief, RFP^+^ NIH3T3 cells and GFP^+^ mESCs were resuspended using a cell electrofusion medium (0.3 mM MgCl_2_; 0.3 mM CaCl_2_; 0.3 M mannitol) (osmolality: ~300 mOsmol/kg, conductivity: 0.012 S/m) at a density of 5×10^5^/ml. Then the mixed cells (1:1) were loaded into microfluidic device using a syringe pump (Harvard Pump 11 Plus Dual Syringe, Harvard Apparatus) at a flow rate of 5 μL/min. At first, a cell alignment signal (frequency, 1 MHz; Vp-p, 3–10 V; time, 1 min) was used to induce cells pairing. Afterwards, a cell electroporation signal (intensity, 9 V; width, 50 μs; interval, 1 s; number of pulses, 3–5) was applied to induce cells electroporation. At last, a cell electrofusion signal (frequency, 1 MHz; Vp-p, 3–10 V; time, 2 min; attenuation of voltage, 50%/min) was added to realize cell fusion. To quantify average ratio of micro-cavities containing paired cells in all micro-cavities, pictures were taken at 10 random locations and more than 300 aggregates in three cell electrofusion chips were examined. Then the percentage of multi-cell pairs, homogeneous cell pairs and electrofused heterogeneous cell-pairs were normalized to all the paired cells. To quantify cell viability, 10 μl cell suspension was mixed with 10 μl Trypan blue (0.04% solution diluted by PBS, Life Technologies) in a slide after electrofusion. Then the number of total cells and viable cells was counted by Automated Cell Counter (Bio-rad). Other cells were centrifuged at 1,000 rpm for 5 min, resuspended with PBS and sorted by flow-cytometry (BD Biosciences).

### Karyotype analysis

Karyotype analysis was performed as described previously [29]. Briefly, cells were incubated with 0.2 mg/ml demecolcine (Sigma-Aldrich) for 3 h. After trypsinization, the cells were resuspended in 0.075 M KCl at 37°C for 30 min. Hypotonic solution-treated cells were fixed in methanol: acetic acid (3:1 in volume) for 30 min, dropped onto precleaned slides and stained with Giemsa (Sigma-Aldrich) for 5 min.

### Reverse transcription (RT)- Quantitative polymerase chain reaction (qPCR)

Total RNA was extracted using an RNA extraction kit (Sangon Biotech) according to the manufacturer’s instructions. Total RNA (approximately 1–2 μg per 20 μl reaction) was reverse transcribed using a RT Reagent Kit (Takara). Quantitative PCR was performed with a Real-Time PCR System (Bio-Rad) using a SYBR Green qPCR Mix (Dongsheng Biotech) according to the manufacturer’s instructions. Relative expression levels were normalized to GAPDH and were calculated using the 2−^ΔΔC(t)^ method. All primers, which were purchased from Sangon Biotech, were listed in Table 1.

**Table 1:** Primers for RT-qPCR.

Name	Sequence
**GAPDH-F**	GTCCCGTAGACAAAATGGTG
**GAPDH-R**	CAATGAAGGGGTCGTTGATG
**OCT4-F**	GCCCGGAAGAGAAAGCGAAC
**OCT4-R**	GCTGATTGGCGATGTGAGTGA
**Nanog-F**	GCCCAGCTGTGTGCACTCAAGG
**Nanog-R**	CACTGGTGCTGAGCCCTTCTGA
**CKAP2-F**	TCTTCTACACCTCGGCTGCAAAGT
**CKAP2-R**	AACTCAGACAACTGCTCCAGGGAA
**LaminA/C-F**	TTTACAGCGAGGAACTGCGTGAGA
**LaminA/C-R**	ATCCAGCTTGGCGGAGTATGTCTT
**Tet1-F**	ACGCTGGAACAAGTGGTAGCCATA
**Tet1-R**	TGAACGTTTGGGTCTTGGAGGTCT
**Tet2-F**	TGCCCTCCCAAGACTCTTCATGTT
**Tet2-R**	GCCCTTTGAATGAATCCAGCAGCA
**Tet3-F**	AACCAGAACGCCAAGGTCAGTAGT
**Tet3-R**	TTGATCTTCTCTGGCGTGCTCAGT

### Western blot

Cells were lysed in ice-cold lysis buffer (Beyotime) containing 1 mM phenylmethylsulfonyl fluoride (PMSF). The lysates were cleared by centrifugation at 15,000 g for 6 min at 4°C. Protein concentrations were determined using a BCA Protein Assay (Beyotime) according to the manufacturer's instructions. Total proteins (100 μg per slot) were electrophoresed on a 5% to 12% SDS-polyacrylamide gradient gel and transferred to PVDF membranes. Then the membranes were blocked in 5% non-fat milk in TBST at 37°C for 30 min. Blots were incubated with antibodies against Nanog (Abcam, 1:1000), against OCT4 (Abcam, 1:1000) or against GAPDH (Proteintech Group, 1:5000) at 4°C overnight. All these antibodies were diluted in blocking buffer. The membranes were washed with TBST, further incubated with horseradish peroxidase (HRP)-conjugated secondary antibody (Beyotime, 1:1000) at 37°C for 1 h, washed with TBST, and detected using an ECL detection system (Thermo Scientific).

### Teratoma formation

Teratoma formation protocol was according to previous report [29]. Briefly, 100 μl of the cell suspension (1×10^6^ cells) was subcutaneously implanted into the inguina of nude mice. Three nude mice were maintained in pathogen-free conditions at the animal facility of Third Military Medical University and received humane care according to the criteria outlined in the ‘‘Guide for the Care and Use of Laboratory Animals” prepared by the National Academy of Sciences. We monitored the tumor size and condition of the mice every week. After 4 weeks, animals were killed with an overdose of sodium pentobarbital (Sigma-Aldrich) and the teratomas were surgically dissected out, fixed with 4% paraformaldehyde in PBS, and embedded in paraffin. Sections were stained with hematoxylin–eosin (HE). The size of teratomas on individual animals was listed in Table 2.

**Table 2:** Size of teratomas.

Number	mESC	Fused cells
**1**	0.32cm^3^	3.6 cm^3^
**2**	0.125 cm^3^	2.16 cm^3^
**3**	0 cm^3^	0.125cm^3^

### Bisulfite DNA methylation analysis

Genomic DNA from NIH3T3 cells, mESCs and fused cells were extracted using the Genomic DNA Kit (TIANGEN). Bisulphite treatment was performed using the DNA Methylation Kit (ZYMO Research). Then the treated DNA were amplified by touchdown PCR. The primers for OCT4 promoter were 5’- TGG GTT TAT TTA TAT TTA GGA TTT TAGA -3’ and 5’- TCT AAA ACC AAA TAT CCA ACC ATAA -3’ (from −483 to −3), and the primers of Nanog promoter were 5’- TAG GAT ATA GGT TTT TTT TTT AGA TTTG -3’ and 5’- AAC ACC AA C CAA ATC AAC CTATC -3’ (from -717 to-187). Touchdown PCR protocol consisted of two phases. In phase 1, PCR was started with initial denaturation at 98°C for 4 min, 20 cycles of denaturation at 94°C for 45 s, annealing at variable temperatures for 45 s, and extension at 72°C for 1 min. The annealing temperature was set at 66°C in the first cycle and, at each of the 19 subsequent cycles, it was decreased by 0.5°C per cycle down to 56°C. Phase 2 consisted of 20 cycles of 94°C for 45 s, 56°C for 45 s, and 72°C for 1 min. Final step was extension of 8 min at 72°C. Then PCR products were cloned into pUcm-T Vector with pUCm-T Vector Cloning Kit (Sangon Biotech) and individually sequenced as previously described [30].

### Dot blot

Analysis DNA samples were denatured at 95°C for 5 min and spotted onto Hybond-N+ nitrocellulose membranes (GE Healthcare). After vacuum-baked at 80°C for 2 h and ultraviolet cross-linking for 15 min, membranes were blocked with 5% non-fat milk in TBST for 1 h and incubated with antibodies against 5mC (Epigentek, 1:1000) and against 5hmC (Active motif, 1:10,000) overnight at 4°C. The antibodies were diluted in blocking buffer. Membranes were washed three times with TBST, further incubated with HRP-conjugated goat anti-mouse secondary antibody (Beyotime, 1:1000) and HRP-conjugated goat anti-rabbit secondary antibody (Beyotime, 1:1000) at 37°C for 1 h respectively. Finally, the membranes were washed with TBST again, and detected using an ECL detection system (Thermo Scientific).

### Glucosylation-coupled Methylation-Sensitive qPCR (GlucMS-qPCR)

GlucMS-qPCR assay was performed as described previously [31]. In brief, Genomic DNA (1 μg) were treated with T4 Phage β-glucosyltransferase (T4-BGT) according to the 5-hmC and 5-mC Analysis Kit (New England Biolabs) instructions. Glucosylated genomic DNA (100 ng) were digested with 10U of either HpaII, MspI or no enzyme (mock digestion) at 37°C overnight, followed by inactivation for 10 min at 95°C. The HpaII and MspI resistant fraction was quantified by qPCR. The primers were designed around at least one HpaII/ MspI site. The resistance to MspI directly translated into the percentage of 5hmC, whereas 5mC level was obtained by subtracting the 5hmC contribution from the total HpaII resistance. All primers which were purchased from Sangon Biotech were listed in Table 3.

**Table 3:** Primers for GlucMS-qPCR.

Name	Sequence	From TSS
**OCT4-F**	ACAGGCTTTGTGGTGCGATG	-272
**OCT4-R**	GGTGGGTGGAGGAGCAGAG	-67

### Immunostaining

Cells on cover slips were fixed with 4% paraformaldehyde for 15 min, permeabilized using 0.1% Triton X-100 in PBS for 15 min and blocked for 60 min in 5% goat serum. For 5mC or 5hmC staining, permeabilized cells were denatured with 2 N HCl for 15 min and neutralized with 100 mM Tris-HCl (pH 8.5) for 10 min before blocking. Primary antibodies against 5mC (Epigentek, 1:500) and 5hmC (Active motif, 1:1000) were diluted in the same blocking buffer and incubated with the samples overnight at 4°C, then incubated with Alexa Fluor 350-labeled Goat Anti-Mouse secondary antibody (Beyotime, 1:500) and Alexa Fluor 350-labeled Goat Anti-Rabbit secondary antibody (Beyotime, 1:500) 1 h at 37°C. Fluorescence images were acquired with a confocal microscopy (Leica).

## Results

### The Microfluidic Electrofusion Chip

To achieve high efficiency cell electrofusion, cell alignment/ pairing controlling was important for the whole process. In our research, DEP force was chosen to conduct cell movement and alignment/ pairing. After a cell alignment signal (AC signal: frequency, 1 MHz; Vp-p, 2–5 V; time, ~2 min), the direction of dielectrophoresis-based cell movement could be predicted by

$$F_{DEP}=2\pi r^{3}\;\varepsilon_{\text{m}}\;\text{Re}[f_{CM}]\nabla {\left|E\right|}^{2},$$

where _m_ was the dielectric permittivity of the medium, E was the electric field strength, and Re[*f*_*CM*_] represented the real part of the Clausius–Mossotti factor with *f*_*CM*_ defined as

$$f_{CM}=\frac{\varepsilon_{c}^{*}-\varepsilon_{m}^{*}}{\varepsilon_{c}^{*}+2\varepsilon_{m}^{*}}.$$

Here $\varepsilon_{c}^{*}$ and $\varepsilon_{m}^{*}$ were the complex permittivity of the cell and medium, respectively. The complex permittivity *ε* ^*^ was given by

$$\varepsilon^{*}=\varepsilon -\frac{j\sigma }{2\pi f},$$

where *f* was the angular frequency of the AC electric field and *σ* was the conductivity. The relative permittivity and conductivity of the electrofusion medium were 78 and 0.012 S/m, respectively [32], whereas these two parameters for cytoplasm were 60 and 0.5 S/m [32, 33]. The corresponding value of Re[*f*_*CM*_] was about 0.88 for 1 MHz AC signal.

Compared with our previous microfluidic chip (shown in the S1 Fig) [27], the new electrofusion chip showed a more desired field distribution concentrated in the fusion zone (micro-cavity/ discrete microelectrode structure) to conduct cell paring and revisable electroporation (Fig 1A), due to the insulated floating silicon between two adjacent microelectrodes. The electric field and transmembrane potential (TMP) distribution in micro-cavity/ discrete microelectrode structure were shown in Fig 1B. The highest electric field located around the electrode, where was also in the micro-cavity structure. In addition, the TMP at point I (the contact point between microelectrode and cell A in Fig 1B) was similar to that at point II (the junction point between cell A and cell B in Fig 1B). And the TMP at these two points were higher than that at point III (the junction point between cell B and cell C in Fig 1B). It was helpful to prevent the first cell (cell A in Fig 1B) from bursting due to the too high TMP and to improve the viability of the fused cells. In addition, the electric field and TMP distribution that were induced by the micro-cavity/ discrete microelectrode structure ensured the elimination of multi-cell electrofusion. The microfluidic chip and micro-cavity/ discrete microelectrode structure were shown in Fig 1C.

### Electrofusion of NIH3T3 cells and mESCs

**Fig 2:**
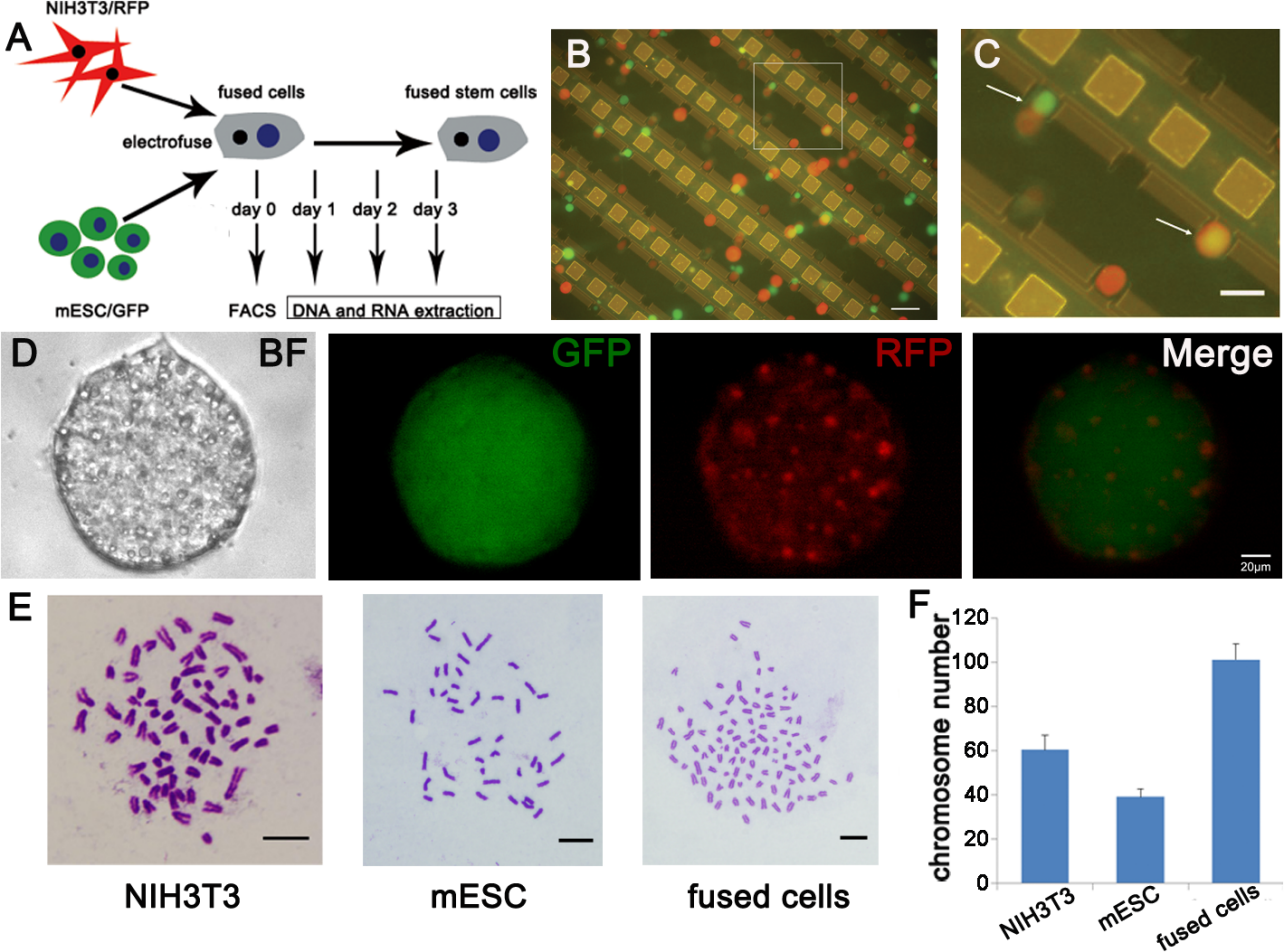
Generation and identification of NIH3T3×mESC fused cells. (A) A schematic representation of fused cells derivation and identification. (B) NIH3T3 cells (red) and mESCs (green) were aligned and fused on the chip. Scale bar, 100 μm. (C) Enlarged picture of the pane in the image B. Arrows indicated cell pairing and fusion. Scale bar, 10 μm. (D) A typical colony of fused cells that were GFP and RFP positive. Scale bar, 20 μm. (E)(F) Karyotype analysis of NIH3T3 cells, mESCs and fused cells. Scale bar, 10 μm.

GFP^+^ mESCs and RFP^+^ NIH3T3 cells were fused by the electrofusion platform. To characterize the fused cells, which were sorted by fluorescence-activated cell sorting (FACS), DNA and RNA were extracted on day 1, 2 and 3 after fusion (Fig 2A). The process of cell pairing and electrofusion were recorded by the micrograph (Fig 2B and 2C). By the DEP force, most cells moved towards the discrete microelectrodes and were trapped in the micro-cavities. Over time, random two cells contacted with each other. In all micro-cavities, 42% (s.d. = 5%) micro-cavities contained paired cells, which consisted of 8% (s.d. = 4%) multi-cell pairs, 47% (s.d. = 8%) homogeneous cell pairs and 45% (s.d. = 9%) pairing mESCs–NIH3T3 cells. Using a cell electroporation signal (intensity, 9 V; width, 50 μs; interval, 1 s; number of pulses, 3–5) and cell electrofusion signal (frequency, 1 MHz; Vp-p, 3–10 V; time, 2 min; attenuation of voltage, 50%/min), 65% (s.d. = 12%) pairing cells were electrofused. By Trypan blue assay, we found that 95% (s.d. = 2%) of cells were viable after the electrofusion. Due to the high integration of the microfluidic chip (over 3.8×10^4^ micro-cavity/ discrete microelectrode structures), we could collect about 5×10^4^ mESCs–NIH3T3 fused cells sorted by FACS after 10 circles of cell electrofusion experiments. Single fused cell could form colony and could express both GFP and RFP (Fig 2D). Besides, karyotype analysis confirmed that the number of chromosomes in fused cells was equal to chromosomes number of NIH3T3 plus that of mESCs (Fig 2E and 2F). It suggested that fused cell derived from NIH3T3 cell and mESC.

### Reprogramming of NIH3T3 cells and pluripotency of fused cells

**Fig 3:**
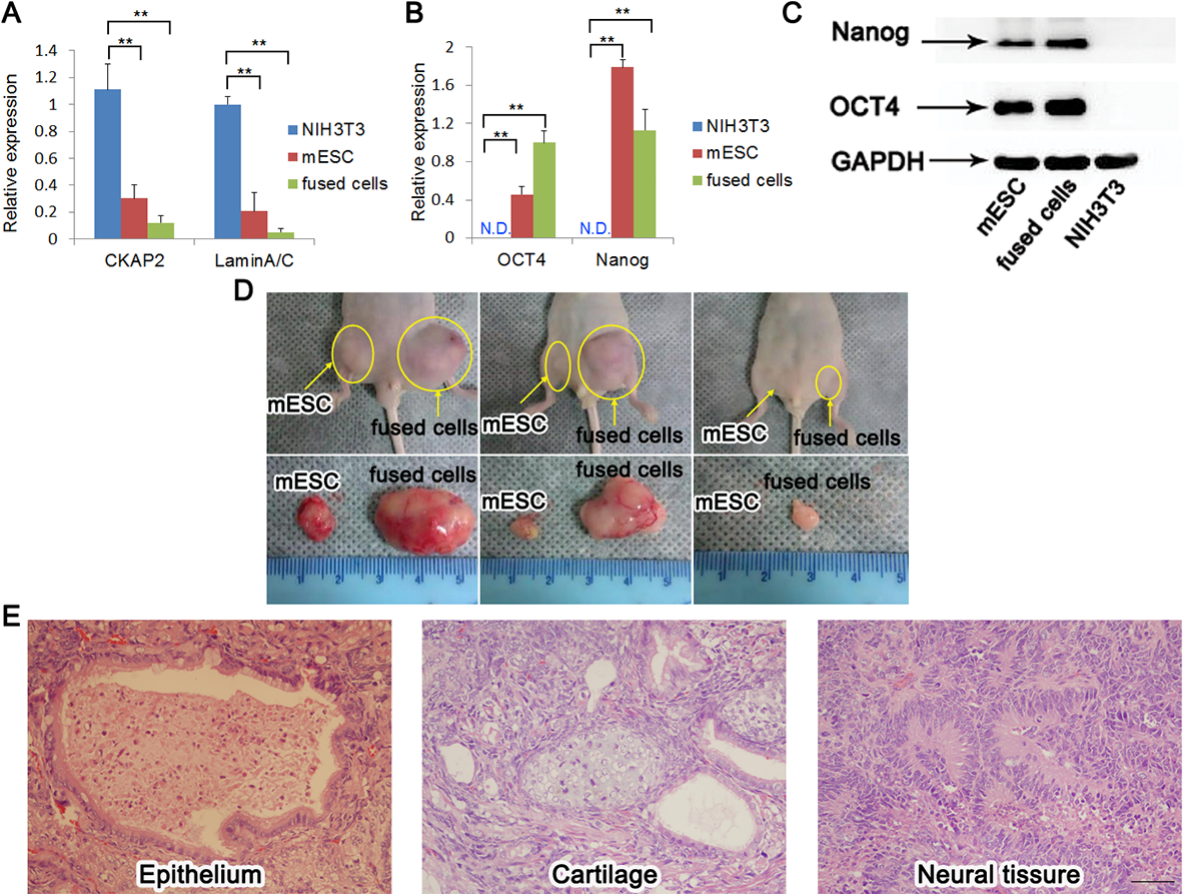
Pluripotency analyses of fused cells. (A) RT-qPCR analysis of the gene expression of somatic cell markers (mean + s.d.; **P<0.01, n = 3). GAPDH was used as an internal control. (B) RT-qPCR analysis of the gene expression of stem cell markers (mean + s.d.; **P<0.01; N.D. indicated no data, n = 3). GAPDH was used as an internal control. (C) Western blotting analysis of the level of Nanog and OCT4. GAPDH was used as a loading control. (D) Teratomas formation in the nude mice. mESCs were used as a control. (E) Histology of teratomas that were derived from fused cells. Left, epithelium (endoderm); middle, cartilage (mesoderm); right, neural tissue (ectoderm). Scale bar, 50 μm.

To characterize the fused cells, we firstly detected the genes expression of fused cells by RT-qPCR assay. It showed that the levels of marker genes for somatic cells, such as CKAP2 and LaminA/C, in fused cells were significantly lower than those in NIH3T3 cells (Fig 3A). In contrast, the levels of the ESC marker genes OCT4 and Nanog were significantly higher in fused cells than those in NIH3T3 cells (Fig 3B). However, there was no significant difference in the levels of these four genes between fused cells and mESCs (Fig 3A and 3B). For protein analysis, western blot assay was performed and showed that both mESCs and fused cells, but not NIH3T3 cells expressed OCT4 and Nanog (Fig 3C). The pluripotency of fused cells was further confirmed by teratoma formation. Fused cells and mESCs were transplanted into nude mice. After four weeks, we observed tumor formation in all the mice. As a control, the tumors of mESCs were smaller than those of fused cells (Fig 3D). HE staining of the teratomas paraffin sections revealed the teratomas contained epithelium, cartilage and neural rosettes, which were representative of endoderm, mesoderm and ectoderm respectively (Fig 3E). It demonstrated that fused cells were pluripotent enough to generate teratomas which contained all three primary layers.

### DNA demethylation in fused cells

**Fig 4:**
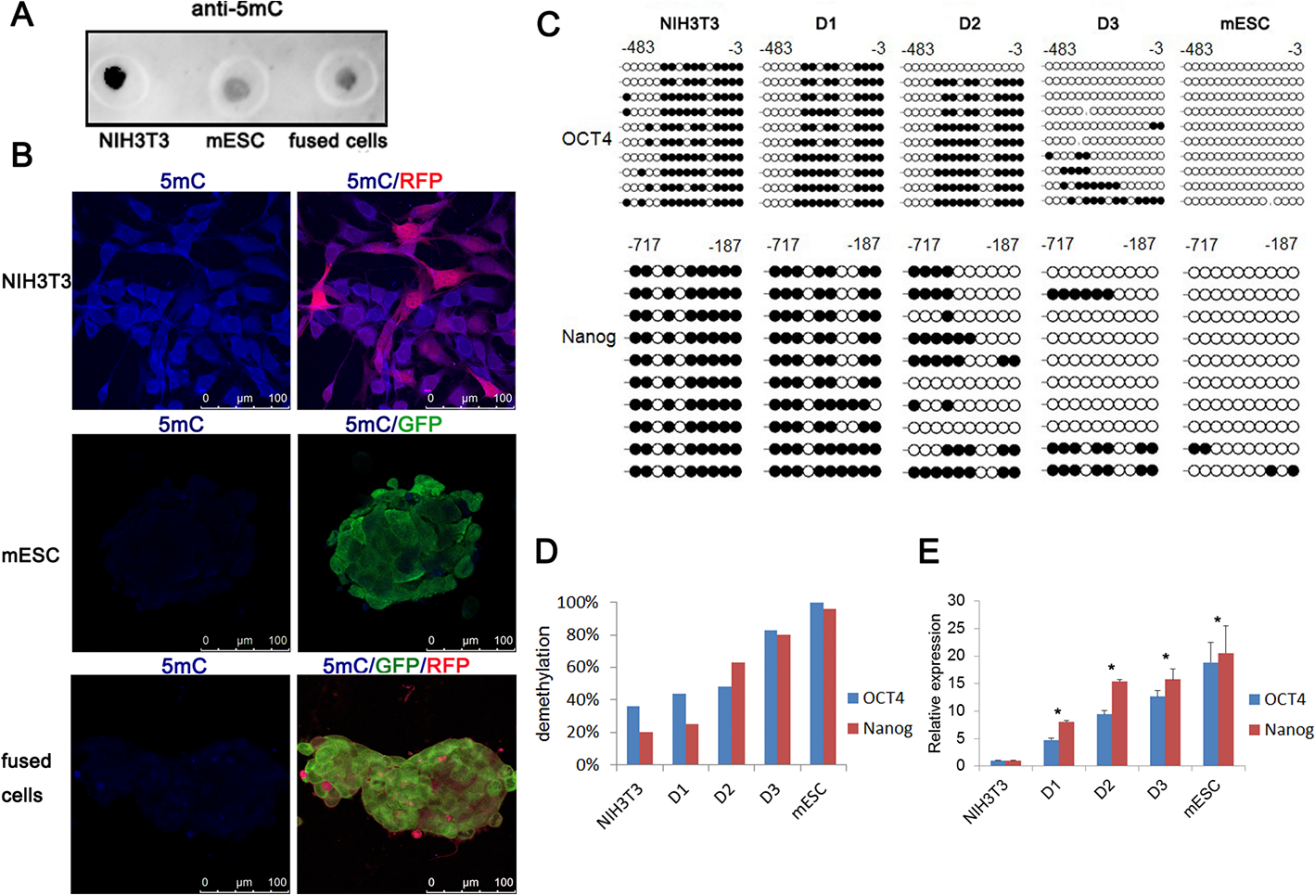
Gradual DNA demethylation and upregulation of pluripotent genes in fused cells. (A) Immunoblotting analysis of 5mC of genomic DNA samples from NIH3T3 cells, mESCs and fused cells at day 3. (B) Immunostaining analysis of 5mC in NIH3T3 cells, mESCs and fused cells at day 3. Scale bar, 100 μm. (C) Bisulphite sequencing analysis of the demethylation status of the OCT4 and Nanog promoters in fused cells on day 1, 2 and 3 after fusion. NIH3T3 cells and mESCs were used as a control. White circles indicated unmethylated CpG dinucleotides, and black circles indicated methylated CpG dinucleotides. (D) The percentage of demethylation at the OCT4 and Nanog promoters assessed by bisulphite sequencing analysis. (E) Time course of OCT4 (blue) and Nanog (red) gene expression in fused cells on days 1, 2 and 3 assessed by RT-qPCR (mean + s.d.; *P<0.05, n = 3). NIH3T3 cells and mESCs served as a control and GAPDH was used as an internal control. D1, D2 and D3 indicated fused cells on day 1, 2 and 3, respectively.

NIH3T3 cells could be reprogrammed into pluripotent stem cells by cell electrofusion. To explore the reprogramming mechanisms, we detected the methylation level in fused cells. A dot blot analysis revealed a marked decrease in 5mC level in fused cells at day 3 compared with NIH3T3 cells. Both mESC and fused cells at day 3 remained hypomethylated (Fig 4A). Consistently, immunostaining also showed a low level of 5mC in fused cells at day 3 and in mESCs, whereas a high level of 5mC was observed in NIH3T3 cells (Fig 4B). Furthermore, we analyzed the time course and the level of demethylation of the OCT4 and Nanog promoters in fused cells. Sequencing of bisulfite-modified DNA showed DNA demethylation of OCT4 and Nanog promoters in fused cells gradually increased through 3 days. On day 3 after electrofusion, demethylation level of the OCT4 and Nanog promoters in fused cells was 83.75% and 80%. As a control, the OCT4 and Nanog promoters in NIH3T3 cells and in mESCs were hypermethylated and hypomethylated, respectively (Fig 4C and 4D). Meanwhile, RT-qPCR analysis showed the time course of OCT4 and Nanog expression in fused cells. By day 1, the expression of OCT4 and Nanog had increased 4.7-fold and 8-fold relative to NIH3T3 cells, respectively. The transcript accumulation of OCT4 and Nanog progressively increased and persisted at 10-fold higher levels on day 3 (Fig 4E). These results indicated that promoters of OCT4 and Nanog genes in fused cells gradually demethylated which paralleled the gradual increase in transcript accumulation.

### 5hmC was involved in reprogramming of fused cells

**Fig 5:**
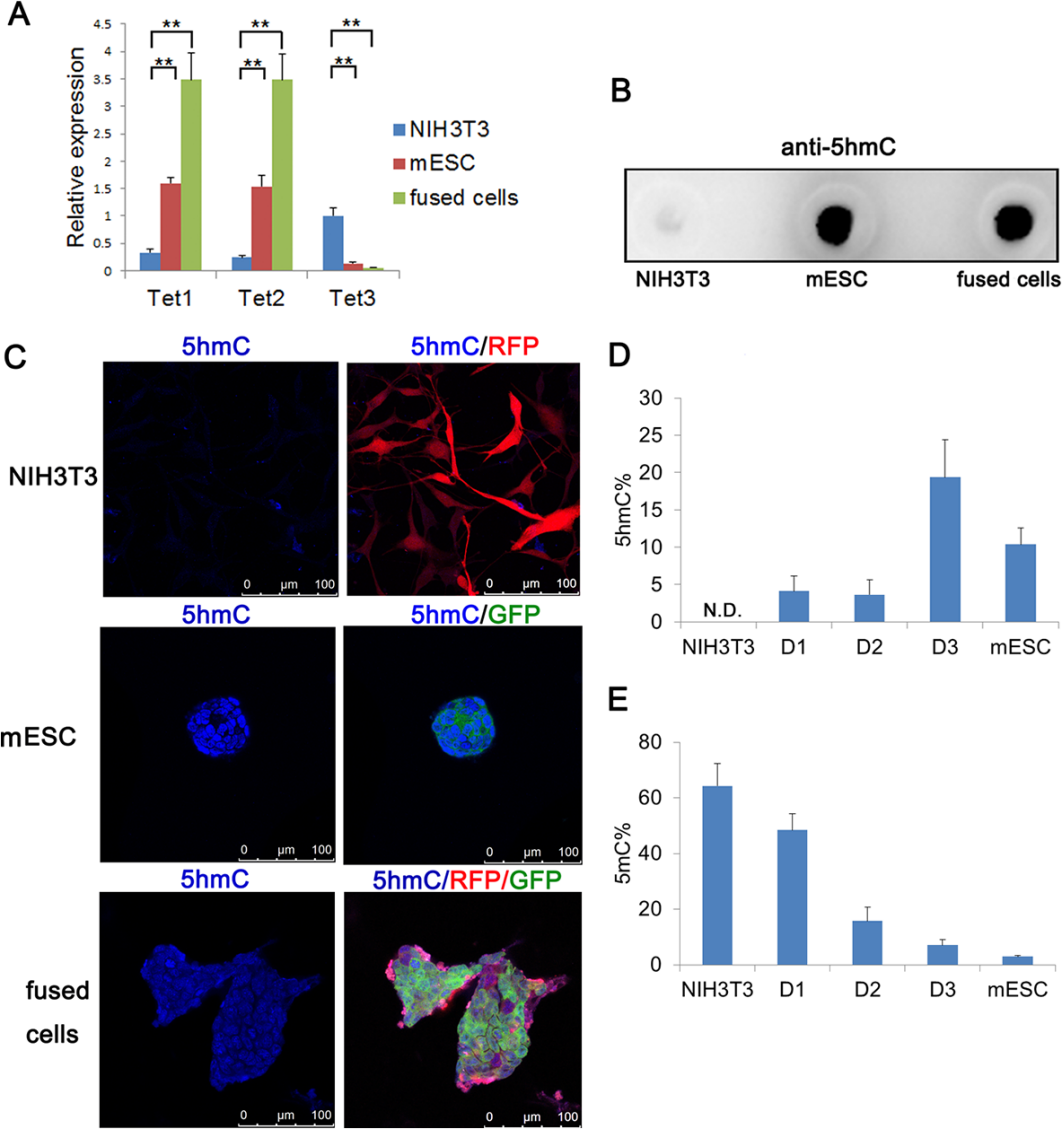
5hmC was involved in DNA demethylation in fused cells. (A) RT-qPCR analysis of the gene expression of Tet enzymes (mean + s.d.; *P<0.05, ** P<0.01, n = 3). (B) Immunoblotting analysis of 5hmC of genomic DNA samples from NIH3T3 cells, mESCs and fused cells at day 3. (C) Immunostaining analysis of 5hmC in NIH3T3 cells, mESCs and fused cells at day 3. Scale bar, 100 μm. (D) (E) Detection of 5hmC and 5mC at OCT4 promoter by glucMS-qPCR (as a percentage of total cytosine). Results were shown as means and s.d. (n = 3). D1, D2 and D3 indicated fused cells on day 1, 2 and 3, respectively.

Recent studies have shown that TET proteins can catalyze the conversion of 5mC to 5hmC, which is involved in DNA demethylation and directly influences genome structure and function [7, 8, 34, 35]. We found that the levels of Tet1 and Tet2 were significantly higher in fused cells compared with those in NIH3T3 (Fig 5A). Tet3 was repressed in fused cells while Tet3 expression was evident in NIH3T3 cells. However, the expression of three TET proteins in fused cells was at comparable levels as that in mESCs. Meanwhile, dot blot assay showed a high level of 5hmC both in mESCs and fused cells at day 3, while a low level of 5hmC was observed in NIH3T3 cells (Fig 5B). Consistently, immunostaining confirmed the similar expressional patterns of 5hmC in fused cells at day 3, mESCs and NIH3T3 cells (Fig 5C). Furthermore, a MspI sensitivity assay (GlucMS-qPCR) was utilized to quantify the 5hmC level at the OCT4 promoter. As expected, 5hmC could barely be detected by GlucMS-qPCR in the OCT promoter of NIH3T3 cells, while mESCs showed 5hmC accumulation in the OCT promoter (Fig 5D). In fused cells, it showed that the level of 5hmC gradually increased through day 3. By day 3, there was a higher level of 5hmC in fused cells than that in mESCs. Besides, GlucMS-qPCR assay also showed that NIH3T3 cells were hypermethylated, mESCs were hypomethylated and fused cells gradually demethylated at the OCT4 promoter, which coincided with prior sequencing results (Fig 5E).

## Discussion

The application of cell electrofusion in the field of reprogramming is limited by technical challenges. However, our research developed an electrofusion chip which was able to induce somatic cells reprogramming. In addition, we found 5hmC was involved in DNA demethylation during the electrofusion mediated reprogramming.

As a DEP-based device, cells have to be resuspended in the electrofusion medium in order to achieve cell electrofusion, which attenuates safety of dielectrophoresis to some extent. The parameters of medium, particularly conductivity and osmolarity, have significant influences to cell electrofusion process. Pucihar et al. has reported that cell survival in experiments involving electropermeabilization can be improved by adjusting the medium conductivity [36]. On the other hand, studies suggest that isotonic medium is beneficial for cells viability when hypotonic buffer significantly improves electrofusion efficiency [37, 38]. In this study, iso-osmotic medium with low conductivity (0.012S/m) was chosen to maximize cells viability. Based on the micro-cavity/ discrete microelectrode structures, our microfluidic chip could electrofuse NIH3T3 and mESCs under a low voltage (9 V), which prevented cell death because of high voltage [27]. In our previous study for NIH3T3-mESCs electrofusion, efficiency of heterogeneous cell paring and cell fusion was 35 ± 9% and 60 ± 28% respectively [27]. However, the efficiency increased to 45 ± 9% and 65 ± 12% in our new chip. After optimizing electric field, we improved electrofusion efficiency and reduced multi-cell electrofusion validated by karyotype analysis [27, 39]. Neither of PEG or traditional electrofusion methods could avoid multi-cell fusion, which would obstruct cell-fusion-mediated reprogramming. However, parent cells were paired randomly in our chip. As a result, we could not generate heterogeneous cell-pairs precisely and a second step of cell sorting using FACS was necessary. Afterwards, we confirmed the pluripotency of the fused cells by detecting genes expression and teratoma formation assay. It could be inferred that somatic cells were reprogrammed when electrofused with pluripotent stem cells, which was similar to PEG and traditional electrofusion methods [14, 40, 41]. Taken together, our microfluidic chip was safe and effective to induce somatic cells reprogramming.

From genome level, the dot blot and immunostaining assays showed NIH3T3 cells were hypermethylated, while fused cells and mESCs were hypomethylated. It indicated that fused cells realized demethylation during reprogramming, which was a key limiting step in somatic cells reprogramming [4, 42]. Furthermore, we focused on the dynamic methylation of ES-cell-specific genes OCT4 and Nanog in fused cells. We found DNA demethylation of OCT4 and Nanog promoters in fused cells increased gradually through 3 days after electrofusion. Compared with PEG mediated reprogramming, the demethylation of OCT promoter ranged in the same level in fused cells. However, the demethylation level of Nanog promoter in fused cells was much higher than PEG mediated reprogramming [4]. Consistently, the up-regulation of OCT4 was alike in fused cells and PEG mediated reprogramming, whereas the up-regulation of Nanog in fused cells was higher than the PEG mediated reprogramming [4]. It suggested that the activation of Nanog was more effective in electrofusion mediated reprogramming.

In the further study on mechanisms of DNA demethylation induced by electrofused, we found upregulated Tet1 and Tet2 and decreased Tet3 in fused cells at day 3 compared with NIH3T3 cells. Consistently, previous researches proved that Tet1 and Tet2 had discrete roles in pluripotent reprogramming and imprint erasure in somatic cells, while Tet3 played a great part in DNA methylation reprogramming processes in the mammalian zygote [6, 43]. From genome level, NIH3T3 could hardly express 5hmC, whereas electrofused cells and mESCs showed a high level of 5hmC. Taken together, we inferred that conversion of 5mC to 5hmC during electrofusion mediated reprogramming was induced by Tet1 and Tet2. What’ more, an increased 5hmC paralleled with a gradually decreased 5mC in OCT4 promoter of fused cells through 3 days after electrofusion. Giving the key role of 5hmC in DNA demethylation, we deduced that 5hmC was involved in DNA demethylation during the reprogramming induced by electrofusion.

To sum up, our electrofusion chip was a better alternative to study reprogramming mechanisms. However, more works should be done to achieve precise heterogeneous cell-pairs.

## Supporting Information

S1 Fig Electric field distribution and cell pairing in the previous microfluidic device with protruding microelectrodes array. (A) Electric field distribution of previous microfluidic device simulated cell trapping and pairing under positive-DEP force. (B) Cell pairing in the previous microfluidic device. Dashed red circle showed cells pairing in the non-fusion zone.
(TIF)
