# Supplementary figures and images for: A Cell Electrofusion Chip for Somatic Cells Reprogramming
Source: PLoS One. 2015 Jul 15;10(7):e0131966. doi: 10.1371/journal.pone.0131966 (PMC4503441; doi:10.1371/journal.pone.0131966)

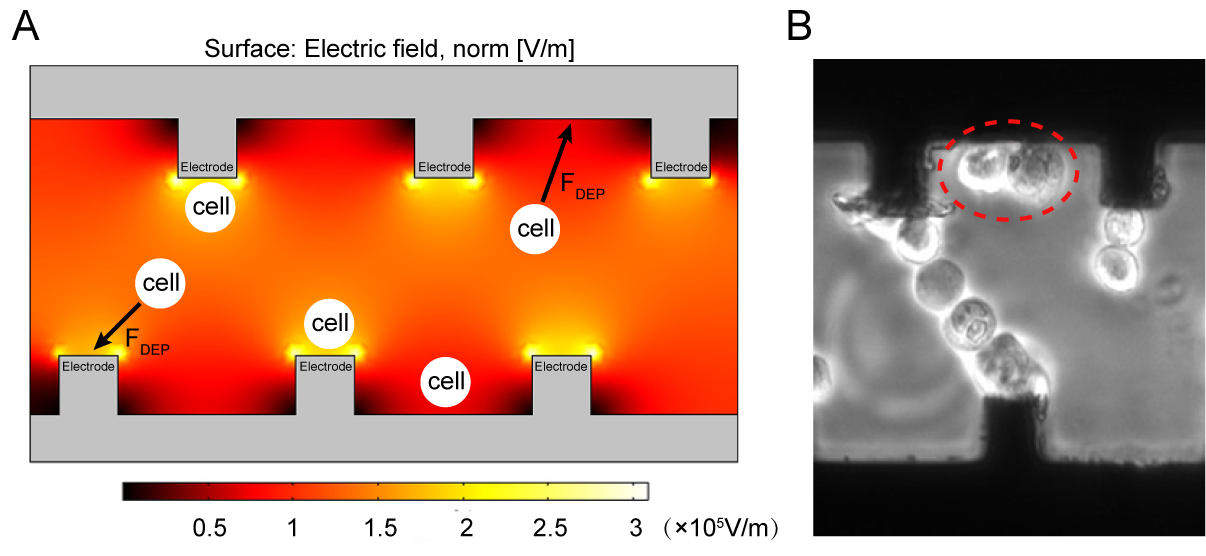

Supplement: S1 Fig — (A) Electric field distribution of previous microfluidic device simulated cell trapping and pairing under positive-DEP force. (B) Cell pairing in the previous microfluidic device. Dashed red circle showed cells pairing in the non-fusion zone. (TIF) [file pone.0131966.s001.tif]
